# Recommendations for defining giant cell arteritis fast-track clinics. English version

**DOI:** 10.1007/s00393-024-01532-9

**Published:** 2024-06-04

**Authors:** Wolfgang A. Schmidt, Michael Czihal, Michael Gernert, Wolfgang Hartung, Bernhard Hellmich, Sarah Ohrndorf, Gabriela Riemekasten, Valentin S. Schäfer, Johannes Strunk, Nils Venhoff

**Affiliations:** 1 Klinik für Innere Medizin, Abteilung Rheumatologie und Klinische Immunologie, Standort Berlin-Buch, Immanuel Krankenhaus Berlin, Lindenberger Weg 19, 13125 Berlin, Germany; 2 Sektion Angiologie—LMU-Klinikum, Medizinische Klinik und Poliklinik IV, München, Germany; 3 Medizinische Klinik 2, Rheumatologie/Klinische Immunologie, Universitätsklinikum Würzburg, Würzburg, Germany; 4 Klinik für Rheumatologie und klinische Immunologie, Asklepios Klinik Bad Abbach, Bad Abbach, Germany; 5 Klinik für Innere Medizin, Rheumatologie, Pneumologie, Nephrologie und Diabetologie, medius Klinik Kirchheim, Kirchheim unter Teck, Germany; 6 Medizinische Klinik mit Schwerpunkt Rheumatologie und Klinische Immunologie, Charité—Universitätsmedizin Berlin, Berlin, Germany; 7 Klinik für Rheumatologie, Universitätsklinikum Schleswig-Holstein, Lübeck, Germany; 8 Sektion Rheumatologie und klinische Immunologie, Medizinische Klinik und Poliklinik III, Universitätsklinikum Bonn, Bonn, Germany; 9 Klinik für Rheumatologie, Krankenhaus Porz am Rhein gGmbH, Köln, Germany; 10 Klinik für Rheumatologie und Klinische Immunologie, Universitätsklinikum Freiburg, Medizinische Fakultät, Albert-Ludwigs-Universität Freiburg, Freiburg, Germany

**Keywords:** Polymyalgia rheumatica, Ultrasound, Magnetic resonance imaging, Computed tomography, Positron emission tomography, Polymyalgia rheumatica, Ultraschall, Magnetresonanztomographie, Computertomographie, Positronenemissionstomographie

## Abstract

A German expert committee recommends defining fast-track clinics (FTC) for the acute diagnosis of giant cell arteritis (GCA) as follows: easy and prompt reachability at least on weekdays, scheduling appointments ideally within 24 h, examination by a specialist with GCA expertise, ≥ 2 experts per FTC, ≥ 50 patients with suspected GCA per year, sonologists with ≥ 300 (≥ 50) temporal and axillary artery examinations, adherence to standard operating procedures, availability of an ≥ 18 (≥ 15) MHz and a lower frequency linear ultrasound probe, and collaboration with partners for neurology and ophthalmology consultations, magnetic resonance imaging (MRI), positron emission tomography-computed tomography (PET-CT, possibly CT), and for temporal artery biopsy.

## Abstract (de)

Eine Expertenkommission empfiehlt, Fast-Track Kliniken (FTK) für die Akutdiagnostik der Riesenzellarteriitis (RZA) folgendermaßen zu definieren: niederschwellige, mindestens werktägliche Erreichbarkeit, Terminvergabe idealerweise innerhalb von 24 h, Untersuchung durch Facharzt mit RZA-Expertise, ≥ 2 Experten pro FTK, ≥ 50 Patienten mit Verdacht auf RZA-Patienten pro Jahr, Ultraschalluntersucher mit ≥ 300 (≥ 50) Untersuchungen von Temporal- und Axillararterien, Befolgung von Standardisierungsanweisungen, Verfügbarkeit eines Schallkopfs ≥ 18 (≥ 15) MHz- und eines niederfrequenteren Linearultraschallkopfs und von Kooperationspartnern für zeitnahe Durchführung von augenärztlichen und neurologischen Untersuchungen, Magnetresonanztomographie (MRT), Positronenemissionstomographie-Computertomographie (PET-CT, ggf. CT) und Temporalarterienbiopsie.

## Introduction

The diagnosis and treatment of primary vasculitides are integral components of rheumatological care. Giant cell arteritis (GCA) represents the most common primary vasculitis. Delayed initiation of therapy often leads to severe complications such as vision loss. Following the introduction of fast-track clinics (FTC), the proportion of permanent blindness in newly diagnosed GCA cases decreased from 19–37% to 2–13% [3, 5, 6, 10]. These recommendations aim to clarify the concept of “FTC” for healthcare provision.

## Current recommendations and care status

Musculoskeletal ultrasound has been a component of rheumatology training in Germany for over 35 years. Ultrasound expertise and quality equipment are widely available in German rheumatology. The new German regulations for rheumatology training, effective since 2022, are the first worldwide to include vascular ultrasound for acute diagnosis of vasculitis [7].

Diagnosis of GCA should involve clinical history and examination supplemented by a confirmatory test. Temporal artery biopsy (TAB) with histology or imaging modalities such as ultrasound, magnetic resonance imaging (MRI), computed tomography (CT), or 18F-fluorodeoxyglucose positron emission tomography (PET) are suitable for confirmation according to current guidelines [9]. New EULAR recommendations advocate for ultrasound of the temporal and axillary arteries as the preferred imaging modality, based on robust evidence supporting its high sensitivity and specificity, widespread availability, low cost, and feasibility in clinical practice [1].

Mostly, patients with suspected GCA already receive prompt rheumatology appointments. Additionally, various centers in Germany offer FTCs with immediate or 1–2 working day appointments, where expert-led structured clinical history, clinical examination, laboratory diagnostics, and specialized vascular ultrasound are conducted. These experts, usually rheumatologists, perform clinical examination and ultrasound concurrently, ensuring accurate diagnosis confirmation or exclusion. Alternatively, collaborations with angiologists (vascular medicine specialists) exist. If diagnosis remains unclear, an additional imaging modality or a TAB is performed. A PubMed search on 18 February 2024 using “giant cell arteritis” and “fast-track clinic” identified 50 publications since 2014, yet no national or international consensus statement defining FTCs precisely.

## Recommendations for FTC requirements

A team of nine rheumatologists and one angiologist from Germany, experienced in GCA diagnosis and treatment, developed recommendations for defining FTCs (Table 1). This process included a web conference, followed by extensive email communication and another web conference with open voting.

**Table 1:** Recommendations for requirements of a GCA fast-track clinic

No.	Requirement	Consensus
1	Easy and rapid contact for appointment scheduling should be available either 24/7 or at least every working day	10/10
2	Appointments should be ideally scheduled within 24 h, or by the next working day at the latest	10/10
3	Examinations should be conducted by a consulting physician with GCA expertise	10/10
4	FTCs should have at least two consulting physicians with GCA expertise available to cover absences such as vacations or sick leave	10/10
5	FTCs should examine at least 50 patients annually with suspected GCA	9/10
6	The examiner should ideally have performed ≥ 300, but at least ≥ 50 ultrasounds of the temporal and axillary arteries	10/10
7	Standard operational procedures (SOP) should be followed	10/10
8	FTCs should maintain at least one linear probe with a B-mode frequency ≥ 15 MHz, preferably a footprint or hockey-stick probe with ≥ 18 MHz, and a linear probe with lower frequencies	10/10
9	FTCs should have collaborative partners from other disciplines for prompt ophthalmological and neurological examinations, MRI, PET/CT (possibly CT), and TAB	10/10

*FTC* Fast-Track Clinic, *GCA* Giant Cell Arteritis, *MRI* Magnetic Resonance Imaging, *PET* Positron Emission Tomography, *CT* Computed Tomography, *TAB* Temporal Artery Biopsy

## Discussion

Ad 1: Contact for suspected GCA cases should be straightforward and rapid for referring physicians, potentially facilitated by the on-duty hospital physician even on weekends, or via phone with qualified personnel during the workweek. If capacity allows, patients with polymyalgia rheumatica (PMR) may also be seen, as subclinical GCA can occur in this population. Recently, it has been recommended that all patients with suspected or recently diagnosed PMR should be considered for specialist evaluation [4].

Ad 2: In cases of high suspicion for GCA, glucocorticoid therapy should be initiated prior to confirmation, with diagnostic evaluation ideally within the first 3 days to ensure diagnostic accuracy. In case of prolonged therapy, efforts should still be made to confirm the diagnosis. Organizational problems must not delay treatment initiation.

Ad 3–6: Expertise in GCA and ultrasound is essential for accurate diagnosis. A high-quality examination is expected with experience exceeding 300 ultrasound examinations [8]. If performed by a trainee, confirmation by a specialist is necessary. The Rheumaakademie (German rheumatology academy) regularly offers DEGUM (German Society for Ultrasound in Medicine)-certified courses on clinical aspects and ultrasound in GCA and PMR.

Ad 7–8: Detailed technical and operational guidelines can be found, among others, in the updated EULAR recommendations [2]. These recommendations should extend to FTC organization, with standardization instructions being particularly important for on-call services.

Ad 9: Collaborations exist in larger hospitals, within the framework of Ambulatory Specialized Care (ASV), or other outpatient networks. PET-CT examinations are reimbursed only in the ASV sector and mostly for privately insured individuals in German outpatient care. TAB is almost exclusively offered in inpatient settings in Germany.

These recommendations provide initial guidance on FTC prerequisites. They can serve as a basis for an implementation process for certification, potentially supported by the German Society for Rheumatology (DGRh).

## Practice implications

- Fast-track clinics for rapid diagnosis of giant cell arteritis (GCA) should be easily accessible and offer appointments within 24 h on weekdays.
- Requirements include medical specialist expertise in GCA and specific ultrasound examinations, adherence to standardization instructions, and adequate ultrasound technology.
- Collaborative partners, including for ophthalmological and neurological examinations, temporal artery biopsy, and other imaging modalities, should be available.

## Declarations

For this article no studies with human participants or animals were performed by any of the authors. All studies mentioned were in accordance with the ethical standards indicated in each case.

The supplement containing this article is not sponsored by industry.

## References

[1] Bosch P, Bond M, Dejaco C et al (2023) Imaging in diagnosis, monitoring and outcome prediction of large vessel vasculitis: a systematic literature review and meta-analysis informing the 2023 update of the EULAR recommendations. RMD Open 9:e3379 37620113 10.1136/rmdopen-2023-003379 PMC10450079

[2] Dejaco C, Ramiro S, Bond M et al (2024) EULAR recommendations for the use of imaging in large vessel vasculitis in clinical practice: 2023 update. Ann Rheum Dis 83;741-751. 10.1136/ard-2023-224543 10.1136/ard-2023-224543 37550004

[3] Diamantopoulos AP, Haugeberg G, Lindland A et al (2016) The fast-track ultrasound clinic for early diagnosis of giant cell arteritis significantly reduces permanent visual impairment: towards a more effective strategy to improve clinical outcome in giant cell arteritis? Rheumatology 55:66–70 26286743 10.1093/rheumatology/kev289

[4] Keller KK, Mukhtyar CB, Nielsen AW et al (2023) Recommendations for early referral of individuals with suspected polymyalgia rheumatica: an initiative from the international giant cell arteritis and polymyalgia rheumatica study group. Ann Rheum Dis Dec. 10.1136/ard-2023-225134 10.1136/ard-2023-225134 38050004

[5] Monti S, Bartoletti A, Bellis E, Delvino P et al (2020) Fast-track ultrasound clinic for the diagnosis of giant cell arteritis changes the prognosis of the disease but not the risk of future relapse. Front Med 7:589794 10.3389/fmed.2020.589794 PMC7753207 33364248

[6] Patil P, Williams M, Maw WW et al (2015) Fast track pathway reduces sight loss in giant cell arteritis: results of a longitudinal observational cohort study. Clin Exp Rheumatol 33(2 Suppl 89):S103–S106 26016758

[7] Pfeil A, Krusche M, Vossen D et al (2021) Model curriculum of the German society for Rheumatology for advanced training in the discipline internal medicine and rheumatology. Z Rheumatol 80(Suppl 2):64–67 (English version) 34605981 10.1007/s00393-021-01080-6 PMC8752525

[8] Schäfer VS, Chrysidis S, Dejaco C et al (2018) Assessing vasculitis in giant cell arteritis by ultrasound: Results of OMERACT patient-based reliability exercises. J Rheumatol 45:1289–1295 29961687 10.3899/jrheum.171428

[9] Schirmer JH, Aries PM, Balzer K et al (2020) S2k Guideline: Management of large vessel vasculitides. Z Rheumatol 79(Suppl 3):67–95 33156399 10.1007/s00393-020-00893-1

[10] Schmidt WA (2018) Ultrasound in the diagnosis and management of giant cell arteritis. Rheumatology 57(Suppl 2):ii22–ii31 29982780 10.1093/rheumatology/kex461

